# Assessment of Benzene-Induced Hematotoxicity Using a Human-Like Hematopoietic Lineage in NOD/Shi-scid/IL-2Rγ^null^ Mice

**DOI:** 10.1371/journal.pone.0050448

**Published:** 2012-12-03

**Authors:** Masayuki Takahashi, Noriyuki Tsujimura, Tomoko Yoshino, Masahito Hosokawa, Kensuke Otsuka, Tadashi Matsunaga, Satoshi Nakasono

**Affiliations:** 1 Biological Environment Sector, Environmental Science Research Laboratory, Central Research Institute of Electric Power Industry, Chiba, Japan; 2 Department of Biotechnology, Tokyo University of Agriculture and Technology, Tokyo, Japan; 3 Radiation Safety Research Center, Central Research Institute of Electric Power Industry, Tokyo, Japan; Indian Institute of Toxicology Research, India

## Abstract

Despite recent advancements, it is still difficult to evaluate *in vivo* responses to toxicants in humans. Development of a system that can mimic the *in vivo* responses of human cells will enable more accurate health risk assessments. A surrogate human hematopoietic lineage can be established in NOD/Shi-scid/IL-2Rγ^null^ (NOG) mice by transplanting human hematopoietic stem/progenitor cells (Hu-NOG mice). Here, we first evaluated the toxic response of human-like hematopoietic lineage in NOG mice to a representative toxic agent, benzene. Flow cytometric analysis showed that benzene caused a significant decrease in the number of human hematopoietic stem/progenitor cells in the bone marrow and the number of human leukocytes in the peripheral blood and hematopoietic organs. Next, we established chimeric mice by transplanting C57BL/6 mouse-derived bone marrow cells into NOG mice (Mo-NOG mice). A comparison of the degree of benzene-induced hematotoxicity in donor-derived hematopoietic lineage cells within Mo-NOG mice indicated that the toxic response of Hu-NOG mice reflected interspecies differences in susceptibilities to benzene. Responses to the toxic effects of benzene were greater in lymphoid cells than in myeloid cells in Mo-NOG and Hu-NOG mice. These findings suggested that Hu-NOG mice may be a powerful *in vivo* tool for assessing hematotoxicity in humans, while accounting for interspecies differences.

## Introduction

Currently, health risk assessment of various factors is evaluated based on results from epidemiologic surveys, animal testing, cytotoxicity studies, or a combination thereof. In epidemiological surveys, the influence on human health can be evaluated directly [1]. However, it is often impossible to perform such surveys, with the exception of surveys addressing a restricted set of factors that offer health benefits, such as pharmaceuticals. Animal testing, which is used as a substitute for epidemiological surveys, allows for quantifiable assessment under controlled conditions [2]. Although experimental animals have been used to assess the risks of various agents, they may not reflect the responses seen in humans. Instead, responses of human cells to potentially toxic agents can be evaluated using cytotoxicity assays [3]. However, in cell culture, it is extremely difficult to establish cell networks that mimic *in vivo* systems. As a result, a safe margin has been applied to health risk assessments to take into consideration the possibility of insufficient evaluation, particularly regarding interspecies differences, though such extrapolation to humans using safe margins occasionally results in overestimation of risks. However, the underestimation of risks by a small safety margin exposes humans to significant danger. Therefore, to perform more accurate health risk assessments, the development of an *in vivo* evaluation system that can reproduce human responses to toxic factors would be an important breakthrough.

For many years, mouse models transgenically expressing human genes [4], [5] or harboring transplanted human cells, tissues, and organs, called humanized mice [6], have been developed to reproduce the responses of human cells *in vivo*. Mice that are humanized by transplantation of human cells are able to establish networks of human cells in their bodies. The available diverse mouse models were developed by transplantation of various types of cells to immunodeficient strains of mice. In cancer research, the biology of human tumor growth, metastasis, and angiogenesis has been evaluated in these mouse models [7], [8], [9]. More recently, by transplanting human hepatocytes into liver-failure immunodeficient mice (uPA/SCID), mice with human livers have been developed for the study of human infectious diseases and metabolism [10], [11]. Moreover, various types of hematopoietic cells can be produced within immunodeficient NOG mice by transplanting human hematopoietic stem cells [12], allowing for the establishment of a functional human-like hematopoietic lineage [13]. These techniques have proven valuable for the *in vivo* study of human hematopoietic stem cell function [14], infectious disease [15], and drug discovery [16], among other research questions. Interspecies differences in responses to toxicants are influenced greatly by the specificity and expression pattern of receptors, metabolic enzymes, and many other molecules. A human-like hematopoietic lineage may mimic the response to toxicants by human cells, and such humanized mice may therefore prove to be powerful tools for health assessment and aid in our evaluation of the hematotoxicity of various factors, while accounting for interspecies differences.

**Figure 1:**
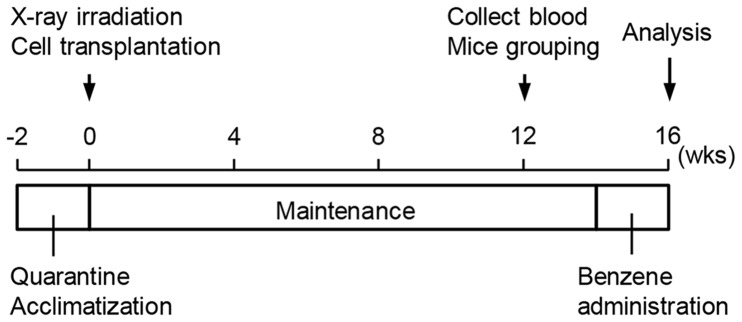
Schematic of the method. After a 2-week quarantine and acclimatization period, human CD34^+^ cells or mouse Lin^−^ bone marrow cells were injected intravenously into irradiated NOG mice. About 4 months after cell transplantation, 0–300 mg/kg-b.w. benzene was administered daily for 2 weeks. The assessment of benzene-induced hematotoxicity was performed using flow cytometric analysis and colony assays.

Hematotoxicity is evaluated according to many factors, including decreased hematopoietic cell counts, abnormal blood coagulation, aberrant myelopoiesis, and induction of leukemia, all of which can be caused by diverse risk factors [17], [18], [19]. Toxicants, such as benzene, can differentially affect human or animal hematopoietic lineages [20], [21]. Here, we took advantage of mice harboring a human-like hematopoietic lineage as a tool for assessing human hematotoxicity *in vivo*. These mice were established by transplanting NOG mice with human CD34^+^ cells (Hu-NOG mice). The response to benzene, a model toxicant, was measured by determining decreases in the number of leukocytes. Furthermore, we established chimeric mice by transplanting C57BL/6 mouse-derived bone marrow cells into NOG mice (Mo-NOG mice). To evaluate whether the response to benzene by Hu-NOG mice reflected interspecies differences, the degrees of benzene-induced hematotoxicities in Mo-NOG and Hu-NOG mice were compared.

**Figure 2:**
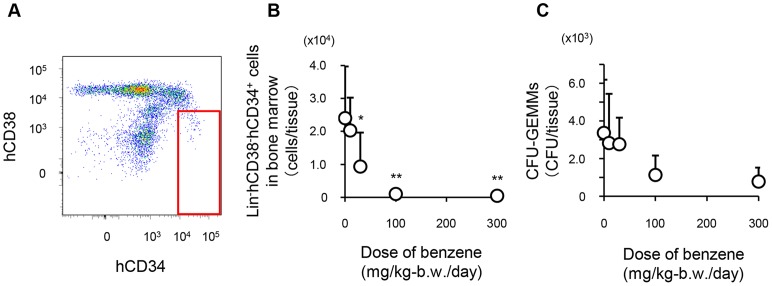
Benzene toxicity in human hematopoietic stem/progenitor cells from Hu-NOG mice. (A) Dot plot of a bone marrow sample from untreated Hu-NOG mice stained with hCD38 and hCD34 within the Lin^−^ gate. (B) Numbers of Lin^−^hCD38^−^hCD34^+^ cells in the bone marrow of Hu-NOG mice after benzene administration (n = 7 or n = 8). (C) Numbers of colony-forming unit-granulocyte/erythroid/macrophage/megakaryocytes (CFU-GEMMs) arising from the bone marrow cells of Hu-NOG mice after benzene administration (n = 6–8). Each point represents the mean ± SD of each group. * *p*<0.05 and ** *p*<0.01 represent significant differences compared with untreated mice, as determined by *t* tests.

## Materials and Methods

### Cells and Mice

Transplanted human CD34^+^ cells isolated from cord blood were purchased from Lonza (Lot: OF4563, Basel, Switzerland) and cryopreserved in liquid nitrogen prior to use. Transplanted mouse Lin^−^ bone marrow cells were prepared from the femurs of 6-week-old C57BL/6J mice. Bone marrow cells were collected by excising and crushing the epiphysis and metaphysis with a mortar and by pushing a needle through the diaphysis. Lin^−^ cells were further purified using a Lineage Cell Depletion Kit (Miltenyi Biotec, Bisley, UK). Fresh Lin^−^ bone marrow cells were used in experiments. As hosts for cell transplantation, immunodeficient NOD/Shi-scid/IL-2Rγ^null^ (NOG) mice (6-week-old, male) were obtained from the Central Institute for Experimental Animals (Kawasaki, Japan). Mice were housed in a specific pathogen-free facility in autoclaved polycarbonate cages and fed sterile food and water *ad libitum*. In addition, NOG mice were maintained on neomycin-polymyxin B in their drinking water.

**Figure 3:**
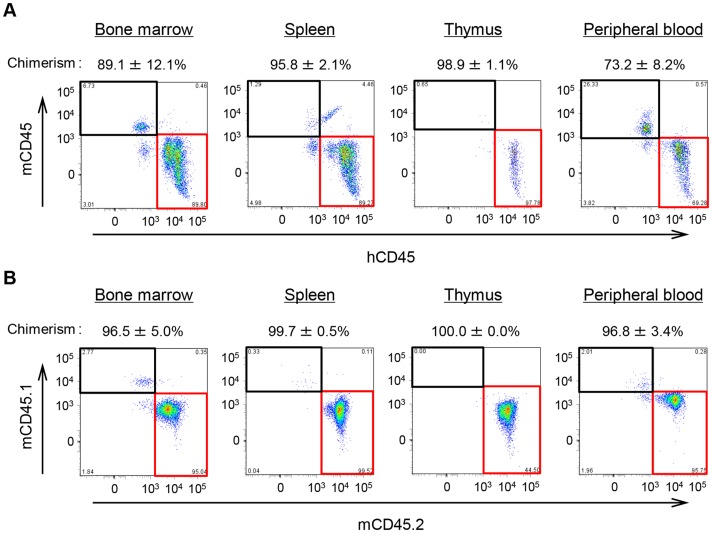
Establishment of hematopoietic cell lineages in NOG mice. Flow cytometric analysis of leukocytes in the peripheral blood and hematopoietic organs of untreated Hu-NOG (A) and Mo-NOG (B) mice. Rates of leukocyte chimerism in Hu-NOG mice were calculated as the percentage of hCD45^+^mCD45^−^ cells in the total CD45^+^ cell population (the sum of human and mouse CD45^+^ cells). Data represent the mean ± standard deviation (SD; n = 7 or n = 8). Rates of leukocyte chimerism in Mo-NOG mice were calculated as the percentage of mCD45.2^+^mCD45.1^−^ cells in the total CD45^+^ cell population (the sum of mCD45.1^+^ and mCD45.2^+^ cells). Data represent the mean ± SD (n = 6–8).

All experimental protocols involving human cells and laboratory mice were reviewed and approved by the Ethical Committee for the Study of Materials from Human Beings and for Research and Welfare of Experimental Animals at the Central Research Institute of Electric Power Industry.

**Figure 4:**
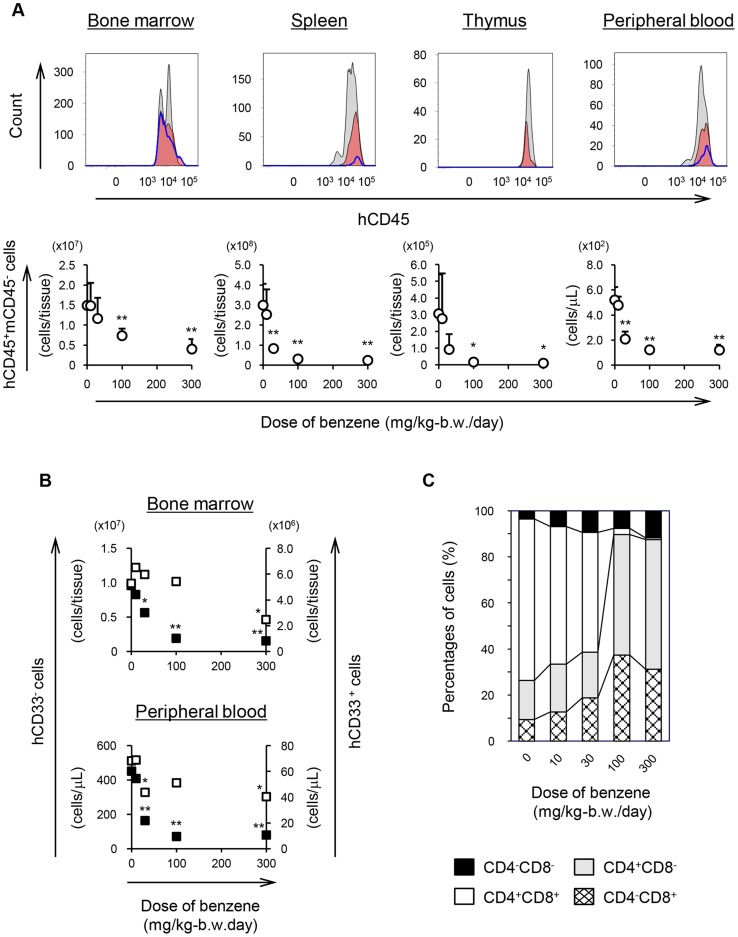
Benzene toxicity in human leukocytes from Hu-NOG mice. (A) Human leukocytes collected from the peripheral blood and hematopoietic organs of Hu-NOG mice. Upper panel: histogram of hCD45^+^mCD45^−^ cells in Hu-NOG mice administered 0 (gray), 30 (red), or 300 mg (blue-lined) benzene/kg-b.w./day. Lower panel: numbers of hCD45^+^mCD45^−^ cells in Hu-NOG mice. Each point represents the mean ± SD of each group (n = 7 or n = 8). * *p*<0.05 and ** *p*<0.01 represent significant differences compared with untreated mice, as determined by *t* tests. (B) Numbers of human myeloid and lymphoid cells in the bone marrow or peripheral blood of Hu-NOG mice. Human myeloid cells were identified as hCD45^+^mCD45^−^hCD33^+^ cells (open square). Human lymphoid cells were identified as hCD45^+^mCD45^−^hCD33^−^ cells (solid square). Each point represents the mean of each group (n = 7 or n = 8). * *p*<0.05 and ** *p*<0.01 represent significant differences compared with untreated mice as determined by *t* tests. (C) The percentage of each T cell population in the thymus of Hu-NOG mice. The value was calculated based on the ratio of hCD45^+^mCD45^−^hCD33^−^ cells. Individual types of T cells were determined by using combinations of anti-hCD4 and hCD8 antibodies. Values represent means (n = 7 or n = 8).

### Cell Transplantation into NOG Mice

After a 2-week quarantine and acclimatization period, whole-body X-ray irradiation of NOG mice was performed at 2.5 Gy using an X-ray generator (MBR-320R, Hitachi Medical, Tokyo, Japan) operated at 300 kV and 10 mA with 1.0-mm aluminum and 0.5-mm copper filters at a dose ratio of 1.5 Gy/min and a focus surface distance of 550 mm. Three to five hours later, the irradiated mice were injected intravenously with human CD34^+^ cells or mouse Lin^−^ bone marrow cells suspended in MEM supplemented with 2% BSA (200 µL containing 4×10^4^ cells per mouse).

**Figure 5:**
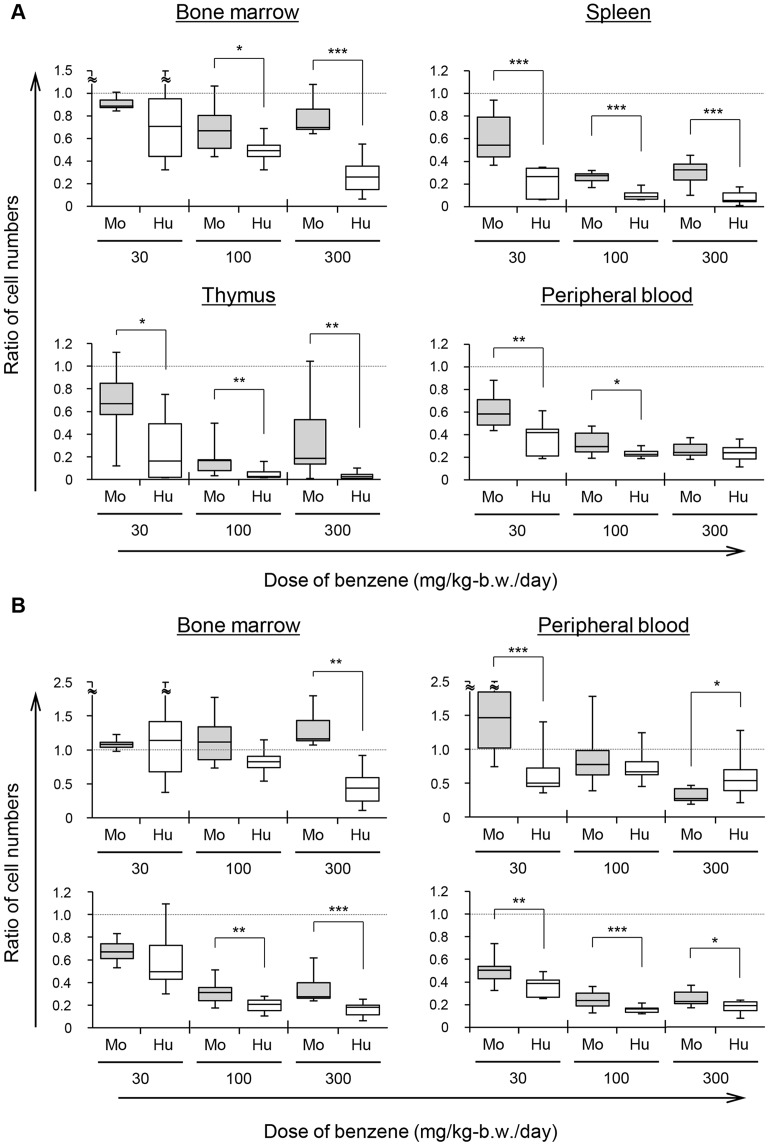
Comparison of benzene toxicity in Hu-NOG and Mo-NOG mice. (A) Ratios of donor cell-derived human or mouse leukocytes in Hu-NOG (Hu) and Mo-NOG (Mo) mice after benzene administration. Each ratio was calculated based on the mean number of leukocytes in untreated Hu-NOG or Mo-NOG mice. (B) Ratios of myeloid (upper) and lymphoid (lower) cells in the bone marrow and peripheral blood of Hu-NOG (Hu) and Mo-NOG (Mo) mice after benzene administration. Each ratio was calculated based on the mean number of myeloid and lymphoid cell in untreated Hu-NOG or Mo-NOG mice. Mouse myeloid cells in Mo-NOG mice were identified as mCD45.2^+^mCD45.1^−^mLy6C/6G^hi/mid^. Mouse lymphoid cells in Mo-NOG mice were identified as mCD45.2^+^mCD45.1^−^mLy6C/6G^lo/−^. The box plot shows the maximum (top of the vertical line), 75th percentile (top of the box), median (middle line in the box), 25th percentile (bottom of the box), and minimum (bottom of vertical line) values of data (n = 6–8). * *p*<0.10 represents marginally significant differences between Hu-NOG and Mo-NOG mice, as determined by Mann-Whitney U tests. ** *p*<0.05 and *** *p*<0.01 represent significant differences.

### Mouse Grouping

Donor human or mouse cell-derived hematopoietic lineages were established in NOG mice by maintenance of the mice for about 3 months after transplantation. For grouping the mice, the properties of the peripheral blood leukocytes of both types of mice were analyzed using a microcavity array system [22], [23], [24] as described previously [22]. Briefly, blood samples (∼20 µL) from the tail vein of transplanted NOG mice were stained with Hoechst 33342 (Life Technologies, Carlsbad, CA) and fluorophore-labeled antibodies. For analysis of Hu-NOG mice, FITC-conjugated anti-hCD45 monoclonal antibodies (mAbs) and PE-conjugated anti-mCD45 mAbs (both from BD Biosciences, San Jose, CA) were used. For analysis of Mo-NOG mice, FITC-conjugated anti-mCD45.2 mAbs and PE-conjugated anti-mCD45.1 mAbs (both from BD Biosciences) were used. Stained blood samples were passed through the microcavities with negative pressure, and only leucocytes were captured. Then, a whole image of the cell array area was obtained using an IN Cell Analyzer 2000 (GE Healthcare Life Sciences, Little Chalfont, UK). The number and rate of host and donor-derived leukocytes was determined from the scanned fluorescence signal of arrayed leukocytes.

On the basis of body weight, the sum of leukocyte counts, and the rates of leukocyte chimerism, Hu-NOG mice were divided into 5 groups of 9–10 mice per group without significant differences between each group. The rates of leukocyte chimerism were calculated as the percentage of donor-derived leukocytes in the total leukocyte population (the sum of donor- and host-derived leukocytes). Mo-NOG mice were divided into 4 groups of 8 mice each.

### Administration of Benzene

Published epidemiological research regarding short-term exposure to benzene has shown that the lowest-observed adverse effect level (LOAEL) of benzene-induced hematotoxicity based on decreasing leukocyte counts in the peripheral blood, is 60 ppm [25]. When inhalation exposure levels are converted into oral administration levels, 60 ppm is equivalent to 30 mg benzene/kg-b.w./day (conversion conditions are as follows: respiratory volume, 20 m^3^/day; absorptivity, 50%; body weight, 70 kg) [26]. Benzene toxicity depends on the amount absorbed and not the site of absorption [26], [27]. Therefore, in the present study, 0, 10, 30, 100, and 300 mg/kg-b.w. benzene, suspended in corn oil, were administered by gavage to Hu-NOG mice daily for 2 weeks, starting at about 4 months after transplantation. In the case of Mo-NOG mice, the amounts of benzene administered were 0, 30, 100, and 300 mg/kg-b.w./day. Because mouse cells have lower susceptibility to benzene than human cells [20], [21], the administration of 10 mg/kg-b.w. benzene to Mo-NOG mice was not performed.

### Cell Preparation from the Peripheral Blood and Hematopoietic Organs

After benzene administration for 2 weeks, samples from the bone marrow, spleen, thymus, and peripheral blood were harvested from each mouse. Bone marrow cells were collected as described above. The spleen and thymus were crushed between 2 glass slides. Peripheral blood was aspirated from the postcava under anticoagulation treatment. Erythrocytes that could have interfered with further evaluation were lysed using VersaLyse (Beckman Coulter, Fullerton, CA). Collected cells were suspended in phosphate buffered saline supplemented with 4 mM EDTA and 0.5% BSA. Mice in which edema was observed in the thymus at the time of dissection were not used for subsequent analysis. We did not observe detectable differences in the appearance of abnormalities or the amount of benzene administered.

### Flow Cytometric Analysis

Hematopoietic cells collected from each tissue, and organs were analyzed by flow cytometry. Cells were stained with fluorophore-conjugated antibodies in BD TruCOUNT Tubes (BD Biosciences) and applied to a flow cytometer to determine cell surface markers and cell numbers simultaneously. FITC-conjugated anti-hCD45 mAbs (BioLegend, San Diego, CA), Lineage cocktail (BD Biosciences), PE-conjugated anti-hCD33 mAbs (BioLegend), anti-hCD38 mAbs (BD Biosciences), PerCP-conjugated anti-mCD45 mAbs (BioLegend), APC-conjugated anti-hCD4 mAbs (BioLegend), anti-hCD34 mAbs (BD Biosciences), and APC-Cy7-conjugated anti-hCD8 mAbs (BioLegend) were used to analyze Hu-NOG mice. FITC-conjugated anti-mLy6C/6G mAbs, PerCP-Cy5.5-conjugated anti-mCD45.2 mAbs, and APC-conjugated anti-mCD45.1 mAbs (all from BD Biosciences) were used to analyze Mo-NOG mice. Flow cytometric analysis was conducted using the FACSCanto II (BD Biosciences) system. A total of 10,000 events were analyzed for each sample. FlowJo software (TreeStar, Ashland, OR) was used for the analysis of flow cytometry data. Data from several samples in which the number of leukocytes exceeding 2 standard deviations of the group mean was detected were not used for analysis.

### Colony-forming Assay

Bone marrow cells (1×10^5^) collected from Hu-NOG mice were plated in methylcellulose-based medium (MethoCult H4034, StemCell Technologies, Vancouver, Canada). After 13 days of cultivation at 37°C in a humidified atmosphere containing 5% CO_2_, the numbers of colony-forming unit-granulocyte/erythroid/macrophage/megakaryocytes (CFU-GEMMs) were enumerated using visible light microscopy.

## Results

### Benzene Toxicity in Human Hematopoietic Stem/progenitor Cells from Hu-NOG Mice

About 4 months after cell transplantation, daily oral administration of 0–300 mg/kg-b.w. benzene was performed in Hu-NOG mice for 2 weeks (Fig. 1). We carried out flow cytometric enumerations of Lin^−^hCD38^−^hCD34^+^ cells contained in the bone marrow of Hu-NOG mice (Fig. 2A), which were highly enriched in the population of human hematopoietic stem/progenitor cells. The number of Lin^−^hCD38^−^hCD34^+^ cells in the bone marrow of Hu-NOG mice decreased depending on the amount of benzene administered (Fig. 2B). Compared with the number of Lin^−^hCD38^−^hCD34^+^ cells in the bone marrow of untreated Hu-NOG mice, the numbers of Lin^−^hCD38^−^hCD34^+^ cells decreased significantly following administration of greater than 30 mg/kg-b.w./day benzene (2.4×10^4^, 2.0×10^4^, 9.3×10^3^, 1.0×10^3^, and 4.7×10^2^ cells/tissue were present after 0, 10, 30, 100, and 300 mg/kg-b.w. benzene administration, respectively). In colony-forming assays for multilineage hematopoietic progenitors, the numbers of CFU-GEMMs appearing in bone marrow cells were reduced depending on the amount of benzene administered (Fig. 2C).

### Benzene Toxicity in Human Leukocytes from Hu-NOG Mice

Human leukocytes were identified in the peripheral blood and hematopoietic organs of Hu-NOG mice by double staining with anti-hCD45 and anti-mCD45 antibodies. By maintenance of the mice for about 4.5 months after cell transplantation, human leukocytes were highly represented in leukocytes contained in all target tissues of Hu-NOG mice (Fig. 3A). The numbers of human leukocytes in Hu-NOG mice without benzene administration were 1.5×10^7^ cells/tissue (bone marrow), 3.0×10^8^ cells/tissue (spleen), 3.1×10^5^ cells/tissue (thymus) and 5.2×10^2^ cells/µL (peripheral blood).

Next, we evaluated the toxic effects of benzene on human leukocytes (hCD45^+^mCD45^−^) in the peripheral blood and hematopoietic organs of Hu-NOG mice. The numbers of human leukocytes in all samples were reduced depending on the amount of benzene administered to the same extent as human hematopoietic stem/progenitor cells in the bone marrow (Fig. 4A). The numbers of human leukocytes in Hu-NOG mice given 30 mg benzene/kg-b.w./day were 0.78- (bone marrow), 0.28- (spleen), 0.30- (thymus), and 0.40-fold (peripheral blood) the number in untreated Hu-NOG mice. The number of cells decreased most drastically in the spleen.

We next analyzed the population of human leukocytes in Hu-NOG mice using anti-hCD33 mAbs and found that benzene administration caused a more dramatic reduction in the number of lymphoid cells (hCD33^−^) than in the number of myeloid cells (hCD33^+^) in the bone marrow and peripheral blood (Fig. 4B). Initially, the spleen and thymus contained only a few myeloid cells (less than 4% of total leukocytes). The percentages of individual types of T cells in the thymus, as identified using differentiation markers, are shown in Figure 4C. The relative abundance of hCD4^+^hCD8^+^ cells was affected by benzene administration to a greater extent than the other 3 T cell populations (hCD4^+^hCD8^+^ cells constituted 70.1, 59.8, 52.1, 2.6, and 0.6% of T cells in the thymus of Hu-NOG mice after 0, 10, 30, 100, and 300 mg/kg-b.w. benzene administration, respectively).

### Comparison of Benzene Toxicity in Hu-NOG and Mo-NOG Mice

In this study, NOG mice (CD45.1) with different strain-derived mouse hematopoietic lineages were established by transplanting Lin^−^ bone marrow cells prepared from C57BL/6 mice (CD45.2). In Mo-NOG mice, C56BL/6 mouse cells succeeded in reconstituting the hematopoietic cell population (Fig. 3B). After benzene administration under the same conditions as for Hu-NOG mice, the degree of benzene-induced hematotoxicity suffered by Mo-NOG mice was compared with that of Hu-NOG mice. Humans are known to be more susceptible to the toxic effects of benzene than mice [20], [21]. The cell number ratio of donor cell-derived human or mouse leukocytes in Hu-NOG and Mo-NOG mice after benzene administration, based on the number of leukocytes in untreated mice, is shown in Figure 5A. This comparison indicated that fewer human leukocytes were present in all target tissues of Hu-NOG mice in comparison with the number of leukocytes present in Mo-NOG mice. The difference in leukocyte number ratios between these mouse groups was large, particularly in the spleen and thymus, where lymphoid cells represented most of the leukocytes. In the bone marrow, the differences tended to vary depending on the amount of benzene administered. In contrast, differences in the peripheral blood followed the reverse tendency. Thus, the difference in cell number ratios was larger in lymphoid cells than in myeloid cells (Fig. 5B). Moreover, 0, 30, and 300 mg benzene/kg-b.w./day was administered to C56BL/6 mice in same manner, and the degree of benzene-induced hematotoxicity of the hematopoietic lineage within C56BL/6 mice was evaluated. The rate of decrease in leukocyte numbers in the peripheral blood and hematopoietic organs of C56BL/6 mice, depending on the amount of benzene, was not significantly different for Mo-NOG mice (*p*>0.10).

## Discussion

Here, we evaluated the toxic response of a human-like hematopoietic lineage established in NOG mice using the hematotoxicant benzene [28], [29], [30]. Benzene-induced hematotoxicity is known to be transmitted by the aryl hydrocarbon receptor (AhR) [31]. Benzene metabolism is mediated by signals transmitted through interactions between AhR and benzene, benzene metabolites, or both, and the resulting benzene metabolites and reactive oxygen species induce cell damage [32], [33]. In hematopoietic cells, the AhR is expressed selectively by immature cells, such as hematopoietic stem/progenitor cells [34], [35], [36]. Therefore, the toxic response of immature cells is the main cause of benzene-induced hematotoxicity [34]. When different amounts of benzene were administered by gavage to Hu-NOG mice, the number of human hematopoietic stem/progenitor cells in the bone marrow was reduced in a dose-dependent manner (Fig. 2). Benzene also affected the numbers of human leukocytes in the peripheral blood and hematopoietic organs (Fig. 4A). Thus, benzene-induced hematotoxicity was detected in a human-like hematopoietic lineage established in NOG mice.

Human lymphoid cells showed higher sensitivity to benzene than myeloid cells in Hu-NOG mice (Fig. 4B). In a previous report on benzene-treated mice [37], the same effects on peripheral blood lymphoid and myeloid cells were observed. Microarray data indicate that benzene downregulates the expression of *MEF2c* [34], which encodes a transcription factor. Mef2c deficiency is associated with profound defects in the production of lymphoid cells and an enhanced myeloid output [38]. Moreover, analysis of the thymic T cell profile of Hu-NOG mice showed that double-positive (DP) pre-T cells were more strongly affected by benzene than T cells at other stages of differentiation (Fig. 4C). It has been reported that only the numbers of DP pre-T cells in the thymus are reduced by 2,3,7,8-tetrachlorodibenzo-p-dioxin (TCDD) [36], [39], and TCDD-induced hematotoxicity is also mediated by AhR signaling [40]. Although the molecular mechanism of benzene toxicity in Hu-NOG mice could not be inferred by these results alone, we did observe a normal response to benzene by Hu-NOG mice harboring a human-like hematopoietic lineage. We conclude, therefore, that the human-like hematopoietic lineage was sensitive to at least 1 hematotoxicant, benzene, and that Hu-NOG mice promise to provide a powerful tool for assessing the *in vivo* response of human hematopoietic cells to known and suspected toxicants. Moreover, Hu-NOG mice can contribute to basic research on human hematopoietic cells, particularly with respect to internal tissues and organs. It is important to note that the LOAEL of benzene-induced hematotoxicity in Hu-NOG mice was approximately equivalent to that established for humans [25].

Sensitivity to benzene differs across species, and humans are more susceptible than mice [20], [21]. The cause of interspecies differences in benzene-induced hematotoxicity likely involves differences in the affinity of benzene and the AhR [41] and the amounts and properties of benzene metabolites [20], [42], [43]; however, this has not been proven. In this study, we established chimeric mice, named Mo-NOG mice, by transplanting C57BL/6 mouse-derived bone marrow cells into NOG mice. Then, we compared the toxic responses of donor cell-derived human and mouse hematopoietic lineage in NOG mice (Fig. 5A). In a previous report, Cai et al. [44] discussed the sensitivity of donor-derived human hematopoietic cells to toxicants by comparison with host-derived immunodeficient mouse cells. However, we are skeptical about this comparison between donor-derived cells and irradiated host cells. In this study, a simple and direct comparison was enabled by equalizing the transplant environment of donor cells. It is also important to note that we used C57BL/6 mice, a strain generally used for toxicity tests. Differences in the benzene sensitivities of donor-derived cells from Hu- and Mo-NOG mice undoubtedly indicated that toxic responses within Hu-NOG mice reflected interspecies differences in benzene-induced hematotoxicity.

The toxicity of benzene in leukocytes in the peripheral blood is induced mainly by benzene metabolites produced in organs such as the liver [45], [46]. Because Hu-NOG and Mo-NOG mice obviously possess the same organs, we predicted that the degree of peripheral blood leukocyte toxicity would be almost the same in both. However, there was a significant difference in the number of peripheral blood leukocytes between Hu-NOG and Mo-NOG mice in response to low levels of benzene. This difference may be attributed to differences in the amounts of cells supplied from the bone marrow, spleen, and thymus. In fact, the difference in the number of leukocytes in Hu-NOG and Mo-NOG mice was most significant in lymphoid organs (Fig. 5B). Moreover, in analyses targeting the bone marrow and peripheral blood, differences in susceptibilities to benzene tended to be greater in lymphoid cells than in myeloid cells. These results suggested that interspecies differences in benzene-induced hematotoxicity are mainly due to differences in toxic responses in lymphoid cells, in the regulation of benzene in lymphoid development, or both. We speculate that there may be interspecies differences in the regulation of *MEF2c* expression by benzene on the basis of the reasons stated above.

In conclusion, a human-like hematopoietic lineage established in NOG mice by transplanting human hematopoietic stem/progenitor cells exhibited human-like susceptibility to at least 1 hematotoxicant, benzene. Hu-NOG and Mo-NOG mice offer a well-defined, reproducible, and easy-to-manipulate *in vivo* system for performing species-specific biochemical analyses of benzene metabolism. We think it is reasonable to assume that Hu-NOG mice will provide a powerful *in vivo* tool for assessing the hematotoxicity of chemical and physical agents on human hematopoietic cells. In the future, the similarities of the hematotoxic responses induced in Hu-NOG mice and humans should be evaluated more carefully by analyzing the detailed toxic response mechanism in Hu-NOG mice. Our strategy may be applicable to the study of other organs [47] and other toxicants as well.
